# Memorandum of Arrangements Proposed by the Minister of Pensions

**Published:** 1920-06-19

**Authors:** 


### Disabled Sailors and Soldiers in Civil Hospitals.

### Memorandum of Arrangements Proposed by the Minister of Pensions.

1. THAT the voluntary hospitals which undertake the work will examine and, if found suitable, treat all disabled sailors and soldiers sent to them by any Local War Pensions Committee on the recommendation of a medical referee or by a medical board or other medical officer appointed by the Ministry of Pensions.

It will rest with the authorities of the hospital to decide after examination of the man whether they accept a patient for treatment.

2. It is understood that if there is any difference of opinion as to the suitability of the case for treatment in the hospital, from whatever cause this disagreement may arise, the hospital authority or medical officer of the hospital will communicate at once with the medical referee, with the Local War Pensions Committee, or with the medical officer of the Ministry of Pensions, who may have sent the case to the hospital, stating the grounds of their objection and suggesting also the treatment advised.

### In-Patient Capitation Rates.

3. The rates payable by the Ministry of Pensions for all such cases as are admitted for in-patient treatment shall be such sums, not exceeding 9s. per day to a fully equipped general or special hospital, or 7s. per day to other hospitals, as may be agreed with the individual hospital.

Where a fully equipped general or special hospital *with a medical school attached* or providing equal educational facilities can show on good evidence that the cost of treatment is reasonably higher in that hospital than 9s. per day, an additional payment equal to the extra cost of treatment, according to the hospital's published statements of account, may be made, provided that the capitation rate for a hospital of this class shall in no case exceed 11s. per day. The foregoing terms will not preclude any arrangement by which the cost of treatment is defrayed at a composition rate per case in place of payment per day if such arrangement should be more convenient.

4. These charges, to be paid to the hospitals by the Ministry of Pensions, shall cover all costs, including any allowance made by the hospital in recognition of medical services.

5. The authorities of the hospital will undertake to keep an accurate record of the treatment of discharged men, to notify the Committee or the Ministry of the admission and discharge of all patients sent by a Local War Pensions Committee or by the Ministry of Pensions, and to enter particulars on the medical record card with a statement as to the general condition of the patient.

The foregoing notifications and particulars to be covered by the inclusive capitation charge made for the attendance and treatment of patients.

6. Where a detailed specialist's report is asked for by the Ministry or by a Regional Commissioner of Medical Services, the hospital shall supply such report for a fee of not exceeding one guinea.

If a hospital is asked for a report by a Local War Pensions Committee the request should be returned to the Committee with an intimation that it should be sent in the first instance to the C.M.S.

7. Where a discharged man is admitted for treatment as an emergency case without authority from the Local War Pensions Committee or the Ministry, the hospital shall communicate without delay with the Commissioner of Medical Services, stating the disease for which treatment is required, with a view of ascertaining whether the case is one on which the charges in paragraph 3 above can be made, and where in such case responsibility is accepted by the Commissioner of Medical Services, payment of all charges shall date back to the date of the admission of the patient.

8. Where the Commissioner of Medical Services notifies a hospital that he himself or a medical officer on his behalf desires to visit the hospital for the purpose of examining an ex-Service patient, the hospital will afford facilities for such examination on the understanding that there will be no interference on the part of the visiting medical officer with the hospital administration or medical treatment.

9. It is proposed that these arrangements shall remain in force for one year, but either party may call for reconsideration of the terms at the end of nine months.

### Out-Patient Capitation Rates.

1. *Orthopædic Clinics.*—1s. 6d. an attendance, not including medical supervision, but all other services; 2s., including medical supervision and all other services.

2. *Special Medical and Surgical Clinics.*—Where such cases as cardiac, tropical disease, aural, and ophthalmic are seen at each attendance by a medical or surgical specialist, 3s. an attendance. This includes all services medical or otherwise.

3. *Officers' Clinics.*—3s. an attendance, not including medical supervision but all other services, which will be arranged and paid for direct by the Ministry.

4. *Neurological Clinics.*—It is not possible to arrange a capitation rate for these, but a rent of 10s. 6d. a session can be paid for the use of room and subordinate staff, etc., the Ministry arranging and paying direct the fees of the medical men.

